# Massive Cerebral Gas Embolism under Discectomy due to Hydrogen Peroxide Irrigation

**DOI:** 10.1155/2015/497340

**Published:** 2015-01-20

**Authors:** Junjie Zhang, Chengliang Zhang, Jianqin Yan

**Affiliations:** ^1^Department of Anesthesiology, Xiangya Hospital, Central South University, Changsha 410008, China; ^2^Department of Cardiothoracic Surgery, Xiangya Hospital, Central South University, Changsha 410008, China

## Abstract

Massive cerebral and spinal gas embolism occurs rarely as a complication of discectomy. We report a 54-year-old female who had undergone a discectomy (L3/4 and L4/5) under epidural anesthesia in a local hospital developed multiple massive gas embolisms. At closure, surgeons irrigated the incision wound with hydrogen peroxide. Soon after the irrigation, the patient suddenly developed tachycardia, hypotension, and rapid oxygen desaturation. Subsequently, patient progressed into unconsciousness and right hemianopsia quadriplegia. Computed tomography (CT) scan showed multiple hypointensity spots around the brain due to cerebral gas embolism, which indicated the pneumoencephalos. The likely mechanism was the absorption of hydrogen peroxide into blood. When the amount of oxygen evolved exceeded its maximal blood solubility, venous embolization occurred. Though the patient was treated with supportive treatments and hyperbaric oxygen, she did not get full recovery and was left with severe long-term cerebral injury.

## 1. Introduction

Hydrogen peroxide is a liquid stabilizer which is commonly used as an irrigant for superficial wounds. Used as a typical disinfectant, solution of hydrogen peroxide typically is low concentration, either 3% or 6%. A rare, but known complication of hydrogen peroxide irrigation is cerebral and spinal gas embolism. Systemic gas embolism has been reported as a complication of various surgical operations and invasive maneuvers and is, more often than not, an iatrogenic complication. We report a patient undergoing discectomy experienced neurological damage from hydrogen peroxide irrigation of surgical wound which was evidenced by brain computed tomography (CT) scan showing multiple cerebral gas embolisms.

## 2. Case Report

**Figure 1:**
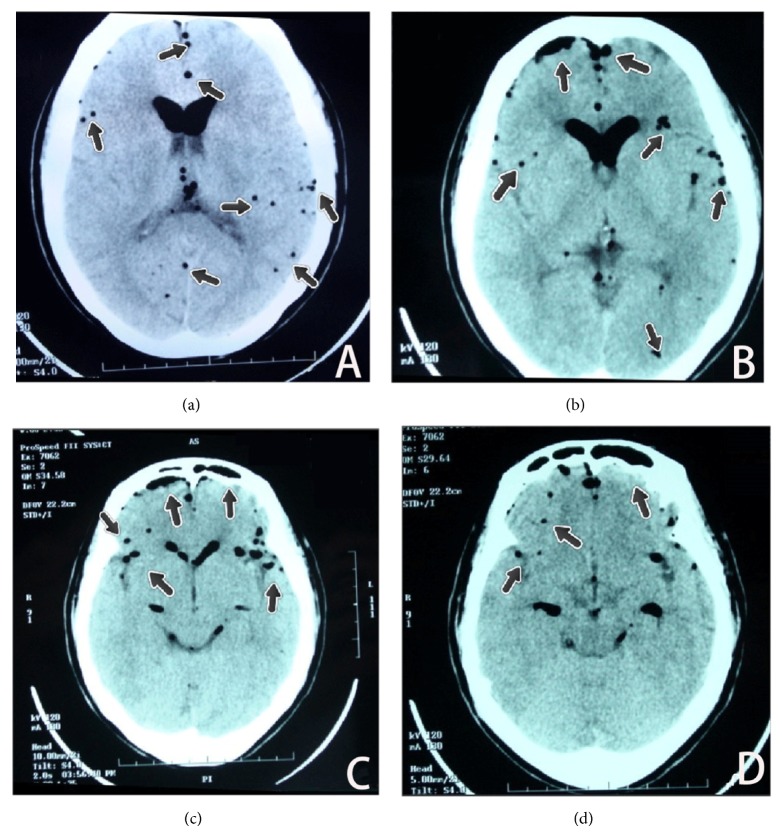
CT of the cerebral immediately after the patient evolved into light coma demonstrating multiple hypointensity spots in cerebral cortex (a), encephalocoele, right frontal lobes (b), right frontal lobes, left anterior temporal lobe (c), and anterior encephalocoele (d).

A 54-year-old right-handed woman underwent discectomy under epidural anesthesia. She had been previously healthy except herniated disks at L3/4 and L4/5 for more than two years. She had no history of stroke or other neurological abnormalities or cardiovascular diseases. The anesthesiologist placed epidural at L1/2 intervertebral space, smoothly with lose of resistance technique. 2% lidocaine 2 mL test dose was administered; there was no sign of spinal anesthesia or intravascular injection. Then 0.5% bupivacaine 6 mL was injected into epidural space with anesthesia level between T10 and L4. After successful anesthesia achieved, patient was placed on prone position. She did not complain of any discomfort during the operation. All the vital signs were normal and patient was conscious till the end of the procedure. Her blood pressure (BP) stayed at 130–110/70–80 mmHg, heart rate (HR) remained 67–80 beats per minute (BPM), and saturation of pulse oxygen (SPO_2_) was 97%. Prior to closure, the surgeons irrigated the incision with 3% hydrogen peroxide 15 mL, then normal saline. Soon after that, patient's HR suddenly stepped up to 110 BPM, while BP declined slightly to 103/68 mmHg and SPO_2_ decreased to 89%. Seeming to lose her consciousness, anesthesiologist immediately gave patient 100% oxygen via mask and 10 mg ephedrine intravenous injection (IV). Then BP slowly returned to 110/70 mmHg, and SPO_2_ returned to 95–97%, while HR still remained at 100–110 BPM. The consciousness of patient deteriorated from indifference to light coma, and Glasgow coma score became equal to 11/15. CT showed multiple massive cerebral gas embolisms as showed in Figure 1. The CT image demonstrated cerebral gas embolism in right frontal lobe. There were multiple hypointensity spots in cerebral cortex. More specifically the abnormalities were seen in the following areas: multiple hypointensity round spots in left posterior temporal lobe, occipital lobe, right anterior temporal lobe (Figure 1(a)), and right frontal lobes (Figure 1(b)); mass gas in the anterior encephalocoele affecting the left greater than the right (Figure 1(c)); right frontal lobes, left anterior temporal lobe (Figure 1(d)). These findings were consistent with multiple areas of cerebral ischemia.

Shortly after operation, the patient dropped into slurred speech, consciousness disturbance, hemiplegia, and right hemianopsia. Quadriplegia was found to coexist with bilaterally dilated pupils. The patient showed difficulties in opening her mouth, problems with her vision and ocular movements and following commands. Her initial vital signs were reported to be abnormal, BP 93/62 mmHg, pulse 84 BPM, body temperature 37.8°C, and respiratory rate 28 per minute. A neurological examination demonstrated a right hemianopsia and a right sided facial drop. The Babinski signs were presented bilaterally, corneal reflection was absent bilaterally, and there were hyperreflexia in upper limbs and absent reflexes in lower limbs. Strength examination showed myasthenia in all muscles, affecting both arms and legs including respiratory muscles, but more serious in the legs at the beginning. During her half-month stay in the hospital after the operation, the patient was under dehydration (25% isosorbide dinitrate 4 mL/kg, iv by drips once a day), neuronutrition (mouse nerve growth factor for injection 30 *μ*g, iv by drips twice daily, and mecobalamin injection 500 *μ*g iv, three times per week), and hyperbaric oxygen treatment twice daily.

After being transferred to our hospital, the patient's symptoms were dyspnea and dysphoria. She was then treated by mechanical ventilation for dyspnea, hypoxia, and possible pulmonary infection. ECG exhibited sinus tachycardia with occasional premature ventricular contractions. She was given lidocaine daily for treating arrhythmia. Chest radiograph demonstrated multiple areas of shadow indicating pulmonary infection or pulmonary infarction secondary to embolism. By day 4 in our hospital, she suffered from subcostal pain. The patient's blood pressure remained hypotensive throughout her hospitalization, which may be due tocerebral gas embolism and dehydration. Under supportive treatment, the patient's vital signs became relatively stable, but her vision and muscle strength was not significantly improved. The patient made little neurological recovery and was left with severe residual symptoms.

## 3. Discussion

Gas embolism is a potentially catastrophe, though uncommon, event which occurs as the consequence of gas entry into the vasculature [1]. Hydrogen peroxide is typically benign with mucosal toxicity being the most commonly reported sign [2]. Direct exposure to hydrogen peroxide may cause cardiorespiratory insufficiency, shock, convulsions, coma, and chemical burns of skin and mucous membranes [3]. Cerebral gas embolism, alone or in combination with other complications, may also develop after absorbtion of a small amount of concentrated hydrogen peroxide [4–10]. Our patient also showed signs of gastritis, which is a potential complication similarly reported in the literature [5, 6].

The suspected source of the crisis was confirmed when the surgeon irrigated the incision with hydrogen peroxide, where mass gas source (appeared pressurized as the incision has a small rift with a large cavity) escaped upon incision of the appendage. There have been three proposed mechanisms by which irrigating hydrogen peroxide could produce a neurological impairment: (A) direct entry of gas into the arterial system; (B) paradoxically, from the venous system through an intracardiac right-to-left shunt, or where intracardiac shunt is excluded, AV malformations in the lungs or overwhelming of the pulmonary capillary filter mechanism; and (C) retrograde cerebral venous gas embolism (RCVGE). Though we did not have direct evidence of images covering the spine, we suspected that O_2_ produced by hydrogen peroxide entered through the ruptured capillary or vertebral venous plexus due to aspiration at the incision site. According to the paraplegia symptom, we believe that gas embolism must have happened on certain site in vertebral artery. Once the gas has crossed into the arterial system, damage occurs when the bubble moves to an end organ and causes ischemia in brain. There are three possible mechanisms of embolization of gas emboli after arriving in the brain. First, as for large gas embolism, the pulmonary capillaries may incompletely filter the O_2_, allowing the O_2_ to move from vein to artery. Oxygen or gas bubbles formed in the venous circulation could pass directly to the arterial circulation through normal circulation. Secondly, oxygen bubbles or undissociated hydrogen peroxide could pass through a pulmonary arteriovenous fistula or undergo transpulmonary transportation over the pulmonary capillary bed [7, 11]. Finally, aspiration could result in hydrogen peroxide itself, or oxygen gas bubbles, being absorbed into the capillary or veins [9].

Diagnosis of a cerebral gas embolism requires high level vigilance. Any neurologic symptoms during the surgery must be investigated. Diagnostic tests contributed to identification of a cerebral gas embolism include arterial blood gas analysis, echocardiography, and chest radiography and computed tomography. CT or magnetic resonance imaging (MRI) may not show the presence of gas in the cerebral vasculature, especially if there is a delay in imaging. Diagnosis of cerebral gas embolism is typically based on a temporal relationship to such known causes as open heart surgery, barotrauma related to scuba diving, suicidal injection of O_2_ [12], or ingestion of hydrogen peroxide [7, 13], or as in this case absorbtion of hydrogen peroxide in the incision. Further supports for the diagnosis come from radiological imaging with noncontrast head CT if gaseous bubbles can be directly visualized in the intracranial circulation [7], or with MRI if diffusion weighted and fluid attenuated inversion recovery (FLAIR) images show multiple areas of cerebral ischemia [4, 9]. Neurologic symptoms may suggest gas embolism of the cerebral and spinal vasculature. Benefit from hyperbaric oxygen therapy is presumed to occur by decreasing the volume of the gas in the intracranial vasculature allowing its redistribution, absorption, and subsequent vascular reperfusion.

In conclusion, the hydrogen peroxide may lower the risk of infection, but absorbtion into the blood vessels on the surgical incision can result in cerebral gas embolism. The occurrence of this life-threatening event confirms the need to pay more attention to the irrigation approaches for antibacterial purpose on surgical site with hydrogen peroxide. Whenever a hydrogen peroxide is used, a high clinical suspicion must be present in any of these patients who display any neurologic symptoms. Prevention, as well as early diagnosis, may decrease morbidity and mortality.
